# An Update Review on *N*-Type Layered Oxyselenide Thermoelectric Materials

**DOI:** 10.3390/ma14143905

**Published:** 2021-07-13

**Authors:** Junqing Zheng, Dongyang Wang, Li-Dong Zhao

**Affiliations:** School of Materials Science and Engineering, Beihang University, Beijing 100191, China; sy1901125@buaa.edu.cn

**Keywords:** thermoelectric, *n*-type oxyselenide, BiCuSeO, Bi_2_O_2_Se, Bi_6_Cu_2_Se_4_O_6_

## Abstract

Compared with traditional thermoelectric materials, layered oxyselenide thermoelectric materials consist of nontoxic and lower-cost elements and have better chemical and thermal stability. Recently, several studies on *n*-type layered oxyselenide thermoelectric materials, including BiCuSeO, Bi_2_O_2_Se and Bi_6_Cu_2_Se_4_O_6_, were reported, which stimulates us to comprehensively summarize these researches. In this short review, we begin with various attempts to realize an *n*-type BiCuSeO system. Then, we summarize several methods to optimize the thermoelectric performance of Bi_2_O_2_Se, including carrier engineering, band engineering, microstructure design, et al. Next, we introduce a new type of layered oxyselenide Bi_6_Cu_2_Se_4_O_6_, and *n*-type transport properties can be obtained through halogen doping. At last, we propose some possible research directions for *n*-type layered oxyselenide thermoelectric materials.

## 1. Introduction

Thermoelectric (TE) materials can achieve the direct transition between heat and electricity without producing other pollutants, providing an effective solution to the energy crisis and environmental problems [1,2]. The dimensionless figure of merit *ZT* defines the efficiency of a thermoelectric device, which derives from three related physical quantities: electrical conductivity (*σ*), Seebeck coefficient (*S*) and thermal conductivity (*κ*), $ZT=\left(\sigma S^{2}T/\kappa \right)$, with absolute temperature *T*. However, the tightly-coupled relationship among these parameters makes it difficult to improve the overall *ZT* [2,3,4,5,6].

Compared with traditional thermoelectric materials, such as Bi_2_Te_3_ [7,8,9,10], PbTe [11,12,13,14,15], SnTe [13,16], MgAgSb [17,18,19], half-Heusler alloys [20,21,22], Zintl phases [23,24], etc., layered oxyselenide thermoelectric materials, mainly including BiCuSeO [2,25], Bi_2_O_2_Se [26,27] and Bi_6_Cu_2_Se_4_O_6_ [28,29,30], consist of earth-abundant, nontoxic, light and lower-cost elements, and have better chemical and thermal stability in the middle temperature range (600–900 K) [2,31]. Therefore, this series was regarded as thermoelectric materials with broad development prospects and studied extensively.

Intrinsic *p*-type semiconductor BiCuSeO possesses a layered ZrCuSiAs structure with space group *P4/nmm* [2,32]. The special layered crystal structure of BiCuSeO is constituted by insulative [Bi_2_O_2_]^2+^ layers and conductive [Cu_2_Se_2_]^2−^ layers heaping along the *c*-axis by turns [33,34]. Due to the weak Van der Waals interaction between layers [9,35,36,37], the large displacement of the Cu atom [38,39] and the heavy Bi atom [3,40,41], BiCuSeO has intrinsically low thermal conductivity [42], which is an inherent advantage as a thermoelectric material. However, most of the reported BiCuSeO are *p*-type semiconductor materials at present, and researches on *n*-type BiCuSeO are relatively few and unsuccessful. The main problems are that no effective electronic dopant was found, and BiCuSeO-based materials with stable *n*-type transport properties have not been obtained yet.

Different from BiCuSeO, the intrinsic Bi_2_O_2_Se exhibits *n*-type transport properties due to a large number of Se vacancies in the crystal structure [43,44]. However, the crystal structure of Bi_2_O_2_Se is very similar to BiCuSeO in which insulative [Bi_2_O_2_]^2+^ layers and conductive [Se]^2−^ layers stack along the *c*-axis alternatively [26,45,46]. Similarly, Bi_2_O_2_Se also has intrinsically low thermal conductivity due to the weak interlayer interaction [26,47,48,49], but its intrinsic carrier concentration (~1.5 × 10^15^ cm^−3^) is too low, resulting in poor electrical transport performance [50,51]. Therefore, current research is mainly focused on increasing the carrier concentration, thus improving the electrical transport performance of Bi_2_O_2_Se.

Bi_6_Cu_2_Se_4_O_6_ is a new type of layered oxyselenide thermoelectric material [28,29,30]. The crystal structure of Bi_6_Cu_2_Se_4_O_6_ can be regarded as a 1:2 ratio of BiCuSeO and Bi_2_O_2_Se heaping along the *c*-axis by turns [28], so there are insulative layers [Bi_2_O_2_]^2+^ and conductive layers [Cu_2_Se_2_]^2−^ and [Se]^2−^ in the structure. The Bi_6_Cu_2_Se_4_O_6_ system not only maintains the low thermal conductivity of BiCuSeO but also can utilize the intrinsic electron carrier concentration of Bi_2_O_2_Se, considered to be a promising *n*-type oxyselenide thermoelectric material. At present, the Bi_6_Cu_2_Se_4_O_6_ system with stable *n*-type transport properties can be obtained by halogen doping [29], and strategies to improve its thermoelectric performance need to be further explored.

**Figure 1:**
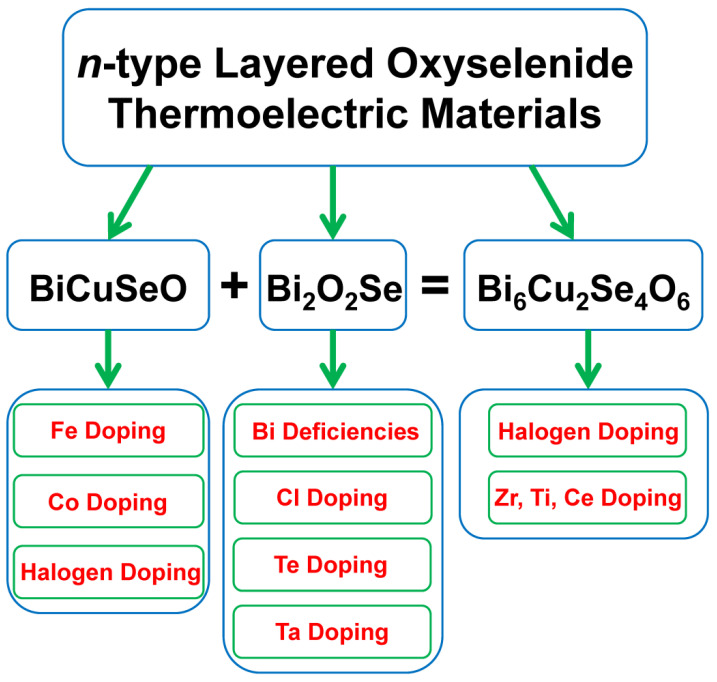
The outline of this review.

Recently, several studies on *n*-type layered oxyselenide thermoelectric materials were reported, which motivates us to systematically summarize the recent progress of these researches. The outline of this review is shown in Figure 1. First, several attempts to realize *n*-type BiCuSeO are summarized. Then, some typical approaches to optimize the thermoelectric performance of Bi_2_O_2_Se are presented. Next, a new type layered oxyselenide Bi_6_Cu_2_Se_4_O_6_ is introduced, and *n*-type transport properties can be obtained through halogen doping and optimized by introducing transition metal elements. At last, some prospective and outlooks were provided for future research in the end.

## 2. Various Attempts to Realize *n*-Type BiCuSeO

### 2.1. Doping Fe at Cu Sites

**Figure 2:**
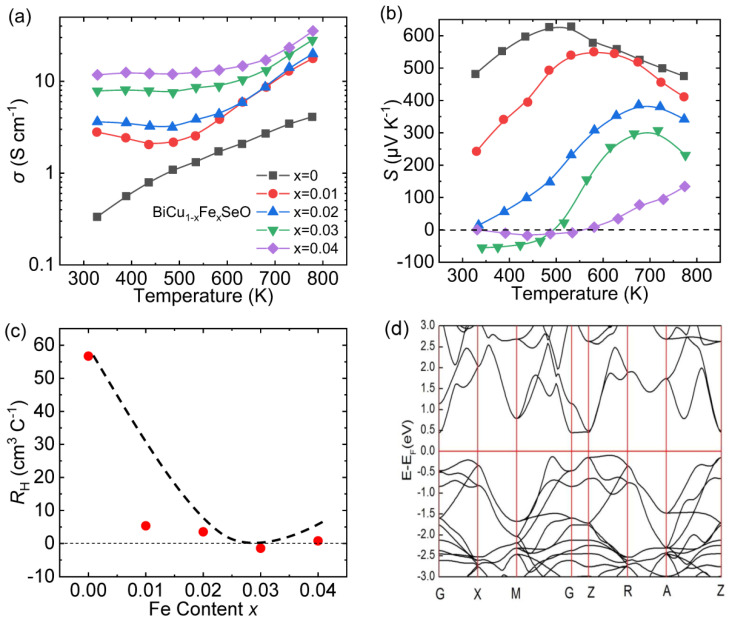
The curves of (**a**) electrical conductivity, and (**b**) Seebeck coefficient varying with temperature for BiCu_1-x_Fe_x_SeO (*x* = 0 to 0.04); (**c**) Fe content dependences of Hall coefficient for BiCu_1-x_Fe_x_SeO (*x* = 0 to 0.04) at room temperature. Data presented in (**a**–**c**) were adopted from Reference [52]. Copyright 2018, The Royal Society of Chemistry. (**d**) Calculated band structure of pristine BiCuSeO near the Fermi level. Copyright, the Royal Society of Chemistry. (**d**) Reproduced with permission from Reference [55]. Copyright 2019, Elsevier Masson SAS.

Pan et al. obtained *n*-type transport properties in the BiCuSeO system in the low-temperature range (300–500 K) through doping Fe at Cu sites [52]. Figure 2a,b display the temperature-dependent electrical conductivity (*σ*) and Seebeck coefficient (*S*) for Fe-doped samples. The *σ* of all samples increases monotonically with temperature, showing semiconductor conduction behavior. Meanwhile, at the same temperature, the *σ* increases with the increasing Fe content, indicating that Fe is an effective dopant for pristine BiCuSeO matrix. The peak values of *S* for samples BiCu_1-x_Fe_x_SeO (*x* = 0 to 0.03) are larger than 150 μV K^−1^ and shift towards higher temperature with the increasing Fe content. The band gap can be estimated by the peak value of *S* through the Goldsmid–Sharp law as Formula (1) [53,54]:

(1) $$E_{g}=2ℯ{\left|S\right|}_{\max}T$$

The peak Seebeck coefficient and corresponding temperature increase with the increase in Fe content, indicating the increase of band gap in those samples. Clearly, *n*-type transport properties can be found in the *x* = 0.03 sample at a low-temperature range, indicating that appropriate Fe content can achieve *n*-type BiCuSeO in a circumscribed temperature range.

The variation of Hall coefficient (*R*_H_) at room temperature is presented in Figure 2c. Expectedly, BiCuSeO-based materials exhibit *p*-type electrical transport properties, and *R*_H_ should be positive. However, the *R*_H_ of BiCu_0.97_Fe_0.03_SeO exhibits a negative value, confirming the *n*-type behavior conjectured by the negative *S*. The transition of electrical transport properties can be explained by the following two formulas [56,57]:

(2) $$R_{H}=\frac{n_{h}\mu_{h}^{2}-n_{e}\mu_{e}^{2}}{e{\left(n_{h}\mu_{h}+n_{e}\mu_{e}\right)}^{2}}$$

(3) $$S=\frac{S_{h}\sigma_{h}+S_{e}\sigma_{e}}{\sigma_{h}+\sigma_{e}}$$

where, subscripts *h* and *e* express hole and electron carriers, respectively. In the above two formulas, *n*_h_ and *n*_e_ are both positive, while *S*_h_ and *S*_e_ are positive and negative, respectively. The decline of *R*_H_ and *S* after doping Fe in Cu sites can be explained by the competition between the intrinsic holes and the electrons introduced by Fe substitution, which indicates that the substitution of Fe for Cu is a kind of donor doping. When the Fe content increases to 0.03, *σ* increases due to the increasing total carrier concentration (*n*_t_ = *n*_h_ + *n*_e_); while *S* declines because the negative contribution of electrons partly counterbalances the positive contribution of holes to *S*, ultimately switching to negative when electrons become majority carriers. Figure 2d exhibits the calculated band structure of pristine BiCuSeO. The valence band maximum (VBM) and conduction band minimum (CBM) are located along the Γ-M and Γ-Z direction, respectively. Larger degeneracy of VBM than CBM leads to a less favorable compromise between large mobility and large effective mass, so the large *S* values are not easy to obtain *n*-type transport.

### 2.2. Doping Co at Cu Sites

Ren et al. aimed to increase the Seebeck coefficient by introducing magnetic ions into the BiCuSeO matrix because previous studies on other systems showed that extra spin entropy could be introduced by the magnetic ions, thereby contributing to increasing the Seebeck coefficient [55]. They found that the BiCuSeO system exhibits *n*-type electrical transport properties below room temperature when the Co content reaches 20%.

**Figure 3:**
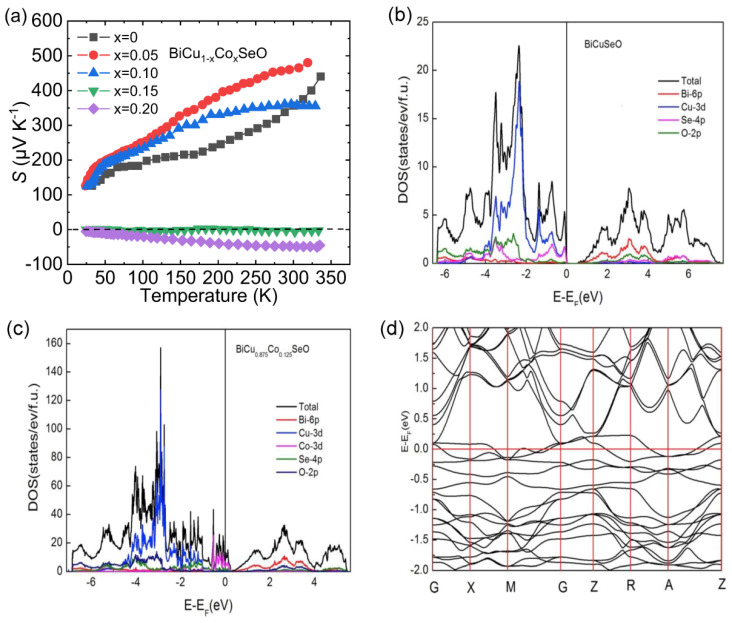
(**a**) The temperature-dependent Seebeck coefficient of BiCu_1-x_Co_x_SeO (*x* = 0.05–0.20). Data were adopted from Reference [55]. Copyright 2019, Elsevier Masson SAS. Calculated density of states (DOS) of (**b**) BiCuSeO and (**c**) BiCu_0.875_Co_0.125_SeO, and (**d**) electronic band structure of BiCu_0.875_Co_0.125_SeO. (**b**–**d**) Reproduced with permission from Reference [55]. Copyright 2019, Elsevier Masson SAS.

Figure 3a shows the temperature-dependent Seebeck coefficient (*S*) for Co-doped samples. For *x* = 0.05 and 0.10 samples, the *S* is positive within the entire temperature boundaries, indicating the dominant carrier is *p*-type. However, when the doping content of Co reaches 15%, the *S* begins to appear negative, indicating that the system exhibits *n*-type electrical transport properties. The negative *S* values of *x* = 0.20 sample within the entire temperature region indicate stable *n*-type electrical transport properties in the BiCuSeO system below room temperature.

The VBM of BiCuSeO mainly comes from the hybridization between Cu 3*d* and Se 4*p* orbitals, while the CBM is derived from the Bi 6*p* orbital [2,58,59], as shown in Figure 3b. For BiCu_0.875_Co_0.125_SeO, the DOS close to Fermi level mainly derives from the Co 3*d* and Se 4*p* orbitals (Figure 3c), and the number of bands crossing the Fermi level is two (Figure 3d), which are both electron cylinder and hole cylinder, indicating the Co substitution introduces *n*-type carriers [60]. It can be seen that Co substitution can change the band structure of BiCuSeO and make the Fermi level reach to the CBM. When the Co content is large enough, electrons become majority carriers.

### 2.3. Doping Halogen (Br, I) at Se Sites

**Figure 4:**
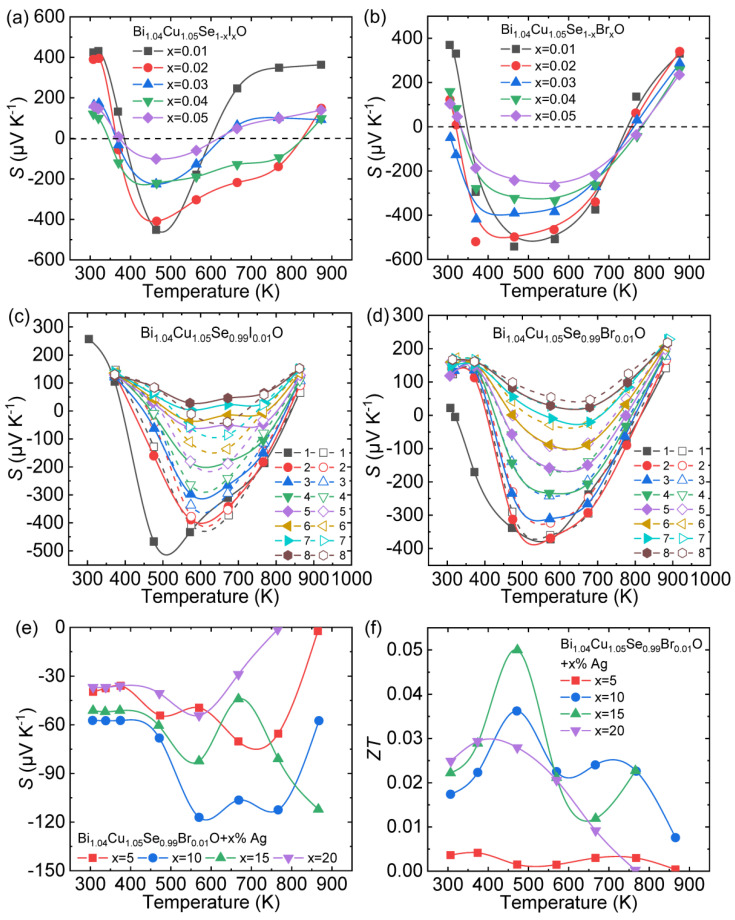
The temperature dependence of Seebeck coefficient for (**a**) Bi_1.04_Cu_1.05_Se_1+x_I_x_O (*x* = 0.01–0.05) and (**b**) Bi_1.04_Cu_1.05_Se_1+x_Br_x_O (*x* = 0.01–0.05). The temperature-dependent Seebeck coefficients obtained through heating-cooling cycle measurements: 8 cycles for (**c**) Bi_1.04_Cu_1.05_Se_0.99_I_0.01_O and (**d**) Bi_1.04_Cu_1.05_Se_0.99_Br_0.01_O, respectively. The solid symbols and lines express the heating process, while the dashed symbols and lines express cooling process. The temperature dependence of (**e**) Seebeck coefficients and (**f**) *ZT* value of Bi_1.04_Cu_1.05_Se_1+x_Br_x_O + *x*% Ag (*x* = 5–20). Data shown in (**a**,**b**,**e**,**f**) were adopted from Reference [61]. Copyright 2017, Elsevier Inc. Data shown in (**c**–**d**) were adopted from Reference [66]. Copyright 2019, Elsevier Ltd and Techna Group S.r.l.

Zhang et al. designed a series of continuous experimental steps to obtain *n*-type BiCuSeO [61]. Firstly, considering that the existence of Bi/Cu vacancies is the main reason for the *p*-type behavior of pristine BiCuSeO [62,63], extra Bi/Cu was introduced into the matrix to fill the vacancies which may produce holes. Finally, the optimal concentrations of extra Bi and Cu are determined as *x* = 0.04 and 0.05, respectively. To increase electron carrier concentration, halogen elements (Br, I) were selected as donor dopants at Se sites and introduced into the Bi_1.04_Cu_1.05_SeO matrix. The *S* as a function of temperature for I/Br doped Bi_1.04_Cu_1.05_SeO samples is presented in Figure 4a,b. The introduction of Br/I can successfully transform Bi_1.04_Cu_1.05_SeO from *p*-type to *n*-type within a certain temperature range, and the negative *S* for I-doped Bi_1.04_Cu_1.05_SeO traverses a narrower temperature range than Br-doped one. In the high-temperature range, *p*-type behavior appears again, indicating that vacancies reproduced as the temperature rises, which may be relevant to the instability of Cu–Br and Cu–I bonds [64,65].

In order to further explore the *p*-*n*-*p*-type behavior in the obtained system, a heating–cooling measurement was carried out for halogen doing BiCuSeO samples [66], as shown in Figure 4c,d. As can be seen, Bi_1.04_Cu_1.05_Se_0.99_X_0.01_O (X = Br, I) changes completely from *n*-type to *p*-type transport behavior after eight heating–cooling cycle measurements. The above results indicate that halogens are effective dopants to obtain *n*-type BiCuSeO but exhibit poor stability. To improve the stability of *n*-type transport, metallic particles were introduced into the halogen-doped Bi_1.04_Cu_1.05_SeO. The temperature dependence of *S* for Bi_1.04_Cu_1.05_Se_0.99_Br_0.01_O + *x*% Ag samples is negative within the entire temperature range, and the maximum |*S*| is ~125 μV/K (Figure 4e). The maximum *ZT* ~0.05 can be reached at 475 K in Bi_1.04_Cu_1.05_Se_0.99_Br_0.01_O + 15% Ag (Figure 4f).

**Figure 5:**
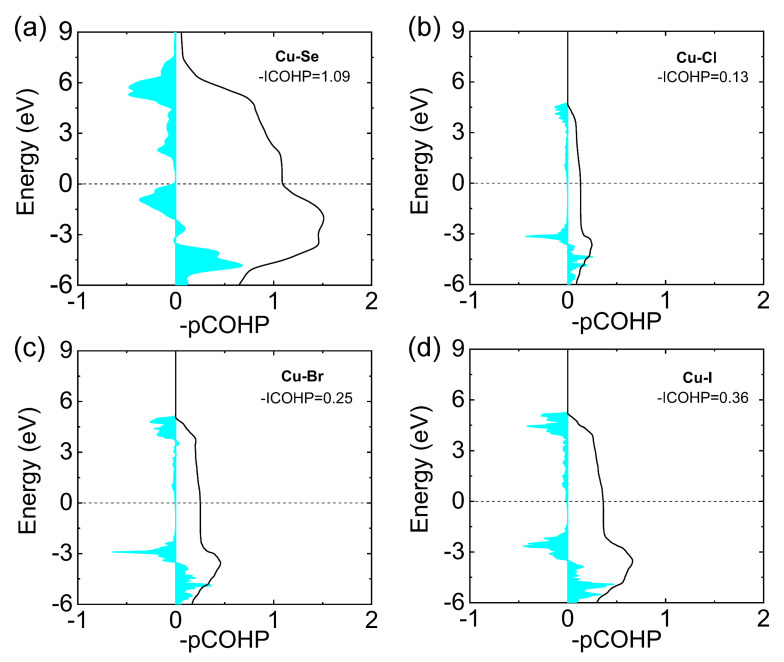
The energy integrated Crystal Orbital Hamiltonian Population (ICOHP) values for (**a**) Cu–Se bonding in BiCuSeO, (**b**) Cu–Cl bonding in Bi_1.04_Cu_1.05_Se_0.99_Cl_0.01_O, (**c**) Cu–Br bonding in Bi_1.04_Cu_1.05_Se_0.99_Br_0.01_O and (**d**) Cu–I bonding in Bi_1.04_Cu_1.05_Se_0.99_I_0.01_O. Data in (**a**–**d**) were adopted from Reference [66]. Copyright 2019, Elsevier Ltd and Techna Group S.r.l.

To further understand the instability of halogen doping in BiCuSeO, the energy integrated Crystal Orbital Hamiltonian Population (ICOHP) values were calculated (Figure 5) [66]. The more negative value of ICOHP indicates the stronger bond strength [67]. As can be seen in Figure 5, after halogen doping, the ICOHP value decreases from ~1.09 eV for Cu–Se to ~0.36 eV for Cu–I, ~0.25 eV for Cu–Br and ~0.13 eV for Cu–Cl, respectively, indicating the weaker bond strength due to halogen doping. The weakened bond strength led to the instability of halogen doping in the BiCuSeO system under the heating–cooling cycle.

## 3. Various Attempts to Enhance Thermoelectric Properties of Bi_2_O_2_Se

### 3.1. Introduce Bi Deficiencies

Due to a large number of Se vacancies in the crystal structure, the pristine Bi_2_O_2_Se exhibits *n*-type semiconductor behavior [43,44]. To improve the conductivity of Bi_2_O_2_Se, thereby optimizing its *ZT*, the general approach is to do donor doping at the Bi/Se sites [47,51,68]. However, Zhan et al. made an innovative attempt that introduces Bi deficiencies into Bi_2_O_2_Se by components deviating from the stoichiometric ratio [46]. In fact, the introduction of Bi deficiencies is equivalent to acceptor doping to the matrix, which runs counter to the general method. However, the increment of Seebeck coefficient and the decrease in thermal conductivity caused by the introduction of Bi deficiencies have optimized the thermoelectric properties of Bi_2_O_2_Se.

**Figure 6:**
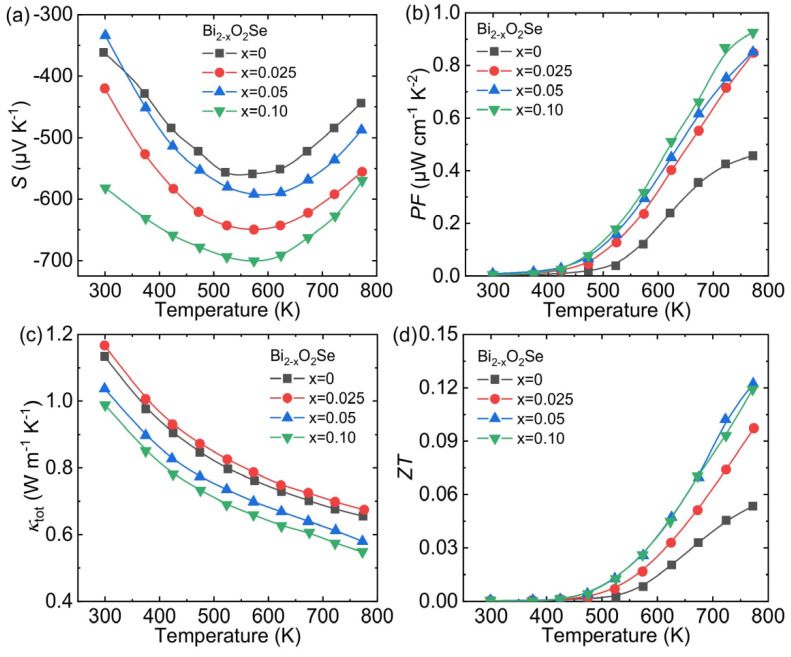
The temperature-dependent thermoelectric transport properties of Bi_2-x_O_2_Se (*x* = 0–0.10): (**a**) Seebeck coefficient, (**b**) power factor, (**c**) total thermal conductivity and (**d**) *ZT*. Data shown in (**a**–**d**) were adopted from Reference [46]. Copyright 2015, The American Ceramic Society.

The introduction of Bi deficiencies has little effect on the electric conductivity (*σ*) of the Bi_2_O_2_Se system, but it can significantly change the Seebeck coefficient (*S*). The absolute values of *S* increased first and then decreased with temperature but always kept a large peak value (−445.6, −556.6, −490.0 and −568.8 μV/K at 773 K, respectively), as shown in Figure 6a. When the carrier concentration in the semiconductor is very low, the *S* can be evinced as the following Formula (4) [4]:

(4) $$S=\frac{8\pi^{2}k_{B}^{2}}{3eh^{2}}m^{*}T{\left(\frac{\pi }{3n}\right)}^{2/3}$$

where *k*_B_, *e*, *h*, m*, *T* and *n* mean Boltzmann constant, electron charge, Plank constant, the effective mass of carrier, the absolute temperature and the carrier concentration, respectively.

The *S* at low temperature increases proportionally with temperature for a given carrier concentration and effective mass. The intrinsic excitation at high temperature is enhanced, and the effective carrier concentration increases, so the *S* decreases. Furthermore, the *S* of Bi deficiencies samples are basically larger than pristine Bi_2_O_2_Se within the entire temperature range, which can be attributed to the influence of m*. Thanks to the significant improvement of *S*, the *PF* peak value reaches ~0.93 μW cm^−1^ K^−2^ at 773 K (Figure 6b), which is twice that of the intrinsic sample (~0.45 μW cm^−1^ K^−2^ at 773 K). The Bi deficiencies strengthen the point defect scattering and lead to a decrease of *κ*_tot_ (Figure 6c). Integrating the enhanced electrical properties and suppressed thermal properties, the peak *ZT* value reaches ~0.12 in Bi_1.9_O_2_Se at 773 K (Figure 6d).

### 3.2. Doping Cl at Se Sites

In the special crystal structure of Bi_2_O_2_Se, the [Se]^2−^ layer is considered to be an electron-conducting pathway [43,44,46]. Therefore, effective electron donor dopants can be used to modify the conductive [Se]^2−^ layer to increase the carrier concentration of the Bi_2_O_2_Se system, thereby enhancing its thermoelectric performance. Tan et al. doped Cl at Se sites and achieved an extraordinary enhancement in the electrical conductivity of the Bi_2_O_2_Se system [51].

**Figure 7:**
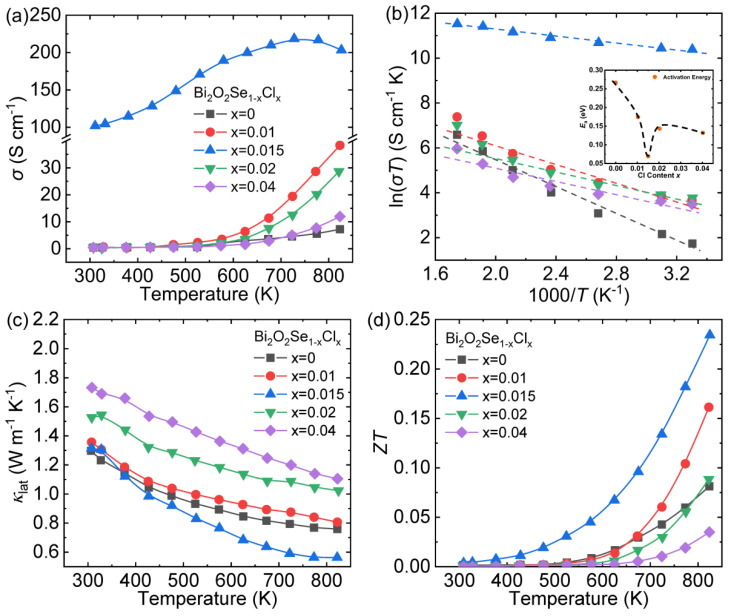
Thermoelectric transport properties of Bi_2_O_2_Se_1-x_Cl_x_ (*x* = 0–0.04): (**a**) electrical conductivity, (**b**) the fitting plots of the small polaron model and the activation energy (*E*_a_) shown in the inset, (**c**) lattice thermal conductivity and (**d**) *ZT*. Data shown in (**a**–**d**) were adopted from Reference [51]. Copyright 2017, The American Ceramic Society.

At room temperature, the *σ* hikes from ~0.019 S cm^−1^ for Bi_2_O_2_Se to ~101.6 S cm^−1^ for Bi_2_O_2_Se_0.985_Cl_0.015_, and then declines obviously as the Cl content increases (Figure 7a). One Cl^−^ doped into the Se^2−^ sites can provide an extra electron, and the measured carrier concentration increased from 1.5 × 10^15^ cm^−3^ to 1.38 × 10^20^ cm^−3^ (*x* = 0.015). However, when the Cl content exceeds the solubility limit, the formation of the low-conductivity second phase Bi_12_O_15_Cl_6_ [69] will reduce the effective doping amount of Cl, thereby deteriorating the *σ*. The small polaron hopping conduction theory was chosen to study the impact of Cl dopant on the *σ*. This theory could be expressed as the following Formula (5) [70]:

(5) $$\sigma =ne\mu =\left(\frac{C}{T}\right)\exp\left(\frac{-E_{a}}{k_{B}T}\right)$$

where *n*, *e*, *μ*, *C*, *k*_B_, *E*_a_ and *T* express the carrier concentration, carrier charge, carrier mobility, the pre-exponential terms, Boltzmann constant, activation energy and the absolute temperature, respectively. Figure 7b shows the linear relationship between ln(*σT*) and 1000/*T*, and the *E*_a_ can be obtained by calculating the slope of the straight line. As shown in the inset of Figure 7b, the *E*_a_ of Cl-doped samples is obviously lower than Bi_2_O_2_Se, indicating that the introduction of Cl is conducive to carrier excitation. In summary, the high *σ* achieved in Cl-doped Bi_2_O_2_Se is estimated to be the result of higher *n* coupled with lower *E*_a_.

Lattice thermal conductivity (*κ*_lat_) of Bi_2_O_2_Se_0.985_Cl_0.015_ declines evidently after 423 K, reaching the lowest value ~0.56 W m^−1^ K^−1^ at 823 K (Figure 7c). This effective decrease in *κ*_lat_ is derived from point defect scattering introduced by Cl substitution coupled with the enhanced grain boundaries scattering. However, for the Bi_2_O_2_Se_0.98_Cl_0.02_ and Bi_2_O_2_Se_0.96_Cl_0.04_ sample, the considerable augment in *κ*_lat_ is the result of the secondary phase Bi_12_O_15_Cl_6_.

Benefitting from both the enhancement of the *σ* and the depression of the *κ*_tot_, the peak *ZT* value ~0.23 at 823 K is achieved in Bi_2_O_2_Se_0.985_Cl_0.015_ (Figure 7d), which demonstrates that Cl is an effective dopant to optimize the thermoelectric performance of Bi_2_O_2_Se.

### 3.3. Doping Te at Se Sites

The *ZT* value of pristine Bi_2_O_2_Se is primarily restricted by the low electric conductivity (~2.0 S cm^−1^) mainly caused by the low carrier concentration (~10^15^ cm^−3^) [50,51]. Fundamentally, this shortcoming can be attributed to the excessively wide band gap (~1.28 eV) [71]. Bi_2_O_2_Te, an isostructure of Bi_2_O_2_Se, possesses a narrow band gap (~0.23 eV) and a moderately high room-temperature carrier concentration (~1.06 × 10^18^ cm^−3^) [45]. Additionally, *p*-type BiCuTeO has a narrower band gap (~0.4 eV) compared with BiCuSeO (~0.8 eV) [72,73], and relevant studies have proved that Te substitution can effectively enhance the electrical conductivity of *p*-type BiCuSeO by narrowing the band gap [74]. Hence, isovalent Te doping at Se sites could be utilized to the *n*-type Bi_2_O_2_Se.

**Figure 8:**
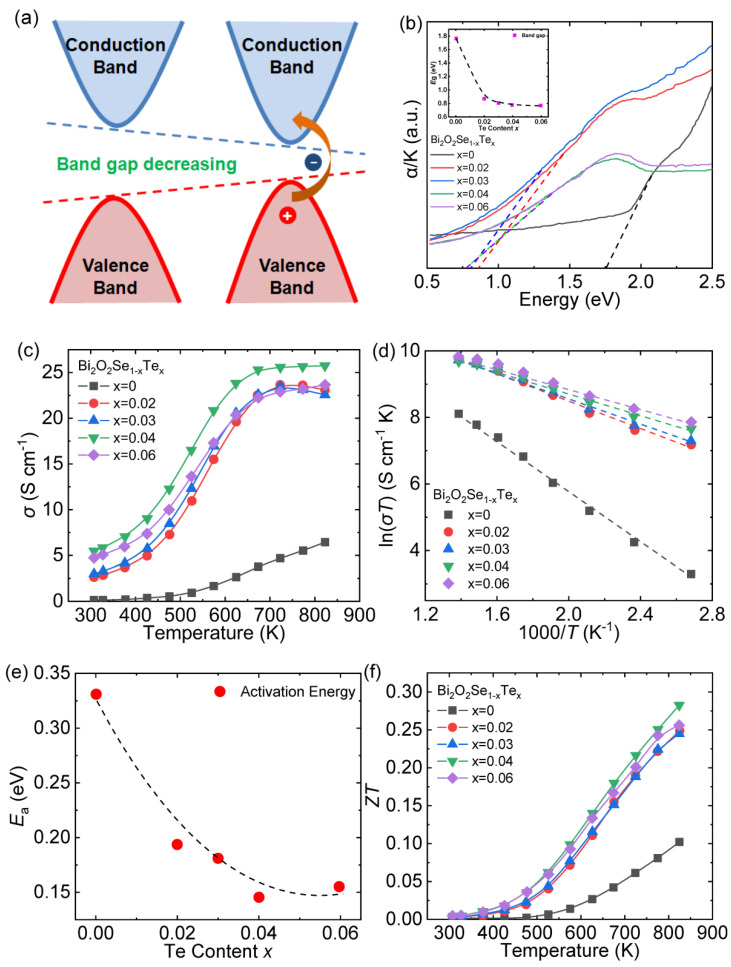
(**a**) The schematic diagram of narrowing the band gap; (**b**) the optical absorption spectra of Bi_2_O_2_Se_1-x_Te_x_ (*x* = 0–0.06) and the band gap varying with Te content shown in the inset; (**c**) electrical conductivity, (**d**) the fitting plot of the electrical conductivity by the small polaron model, (**e**) the activation energy (*E*_a_) and (**f**) *ZT* of Bi_2_O_2_Se_1-x_Te_x_ (*x* = 0–0.06). Data shown in (**b**–**f**) were adopted from Reference [44]. Copyright 2017, The American Ceramic Society.

The significantly narrowed band gap is conducive for electrons to jump across the band gap and enter the valence band (Figure 8a) so that more electrons can be excited and participate in the electrical transport. The measured optical absorption spectrum of Bi_2_O_2_Se_1-x_Te_x_ (*x* = 0.02, 0.03, 0.04, 0.06) samples indicates that the band gap is monotonically reduced from ~1.77 eV for Bi_2_O_2_Se to ~0.78 eV for Bi_2_O_2_Se_0.94_Te_0.06_ with the increasing Te content (Figure 8b) [44]. Considering the apparent difference of the band gap between Bi_2_O_2_Se (~1.77 eV) and Bi_2_O_2_Te (~0.23 eV), the band gap engineering can be effectively tuned by a small amount of Te substitution.

Ultimately, the low carrier concentration of pristine Bi_2_O_2_Se (~10^15^ cm^−3^) was boosted to ~10^18^ cm^−3^, which was increased by three orders of magnitude [44]. The greatly increased carrier concentration makes the *σ* of all the Te-doped samples significantly larger than pristine Bi_2_O_2_Se throughout the entire test temperature range (Figure 8c).

To obtain insight into the electrical transport behavior, the small polaron hopping conduction theory mentioned in the previous work [70] was selected to analyze the electric conductivity. The strong linear correlation between ln(*σT*) and 1000/*T* is exhibited in Figure 8d, and the curve of calculated activation energy *E*_a_ varying with the Te content is depicted in Figure 8e. The *E*_a_ of electronic conduction declines with the increasing Te content, indicating that the Te substitution is beneficial for the intrinsic excitation of electrons, thereby contributing to the optimized *σ*. Figure 8f plots the temperature dependence of *ZT*. Due to the increment in *σ* caused by the narrowing band gap, the thermoelectric performance of Bi_2_O_2_Se was enhanced. Ultimately, the highest *ZT* reaches ~0.28 at 823 K for Bi_2_O_2_Se_0.96_Te_0.04_.

### 3.4. Doping Ta at Bi Sites

Choosing a suitable dopant to enhance its low carrier concentration has always been an important means to optimize the thermoelectric performance of the Bi_2_O_2_Se system. A pentavalent Ta^5+^ cation doping at Bi sites will provide two extra electrons for the matrix. Moreover, Ta is less electronegative than Bi, thereby easily extracting electrons. Therefore, Tan et al. chose Ta as the dopant to increase the carrier concentration, thereby enhancing the electrical transport properties of Bi_2_O_2_Se [68].

**Figure 9:**
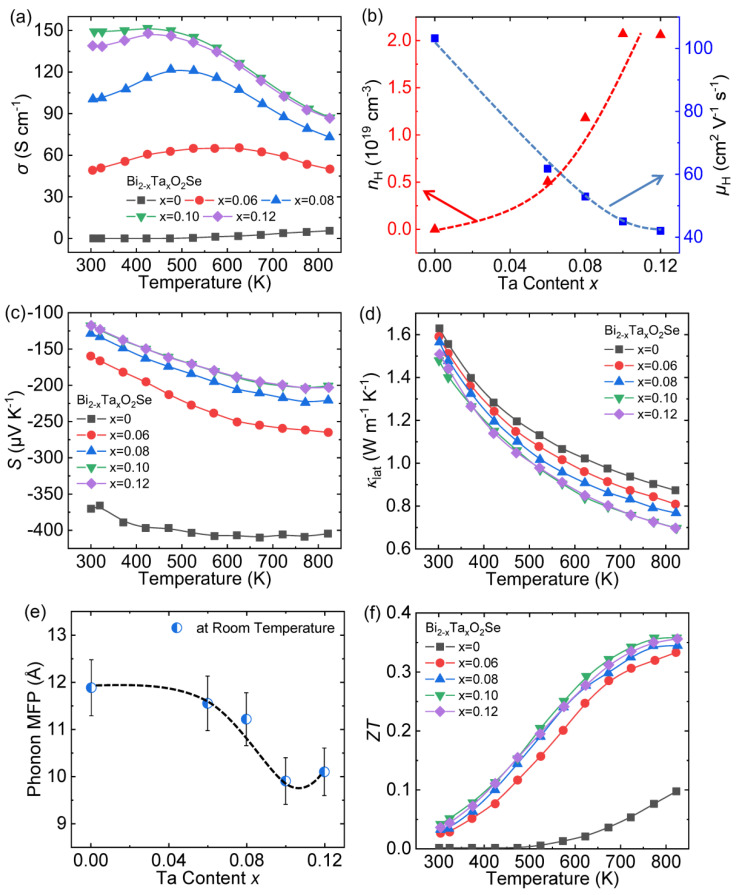
Thermoelectric transport properties of Bi_2-x_Ta_x_O_2_Se (*x* = 0–0.12): (**a**) electrical conductivity, (**b**) the carrier concentration and mobility at room temperature, (**c**) Seebeck coefficient, (**d**) lattice thermal conductivity, (**e**) phonon mean free path (MPF, *l*_ph_) at room temperature, and (**f**) *ZT*. Data shown in (**a**–**f**) were adopted from Reference [68]. Copyright 2019, WILEY-VCH Verlag GmbH & Co. KGaA, Weinheim.

Ta doping can significantly increase the *σ* of Bi_2_O_2_Se, from ~0.02 S cm^−1^ of pristine Bi_2_O_2_Se to ~149.3 S cm^−1^ of Bi_1.90_Ta_0.10_O_2_Se at room temperature (Figure 9a). Meanwhile, the temperature-dependent *σ* transform from semiconductor behavior to mixed-conducting behavior, and finally, Bi_1.90_Ta_0.10_O_2_Se and Bi_1.88_Ta_0.12_O_2_Se even exhibit degenerate semiconductor behavior. The carrier concentration (*n*_H_) and mobility (*μ*_H_) were measured and exhibited in Figure 9b. Ta doping increases the *n*_H_ by four orders of magnitude, from ~10^15^ cm^−3^ to ~10^19^ cm^−3^. In addition to the reason that Ta replaces Bi to provide extra electrons, authors believe that the formation of the Ta_2_O_5_ phase will introduce oxygen vacancies into the matrix, and each oxygen vacancy is compensated by two electrons as the following formula:

$$V_{O}^{\times }\to V_{O}^{\cdot \cdot }+2e^{\prime }$$

Therefore, both Ta doping and oxygen vacancies lead to the increment of *n*_H_. Simultaneously, the stable deterioration of *μ*_H_ implies gradually reinforced carrier scattering, but relatively high *μ*_H_ (>40 cm^2^ V^−1^ s^−1^) can be maintained, which is because performing Ta at Bi sites would not introduce lattice defects into the conductive [Se]^2−^ layers.

Compared with pristine Bi_2_O_2_Se, the absolute value of *S* decreases when the Ta content increases (Figure 9c), which is coincident with the increase in *n*_H_. The calculated weighted mobility (µm^*3/2^) is greatly increased from ~5.41 *m*_0_^3/2^ cm^2^ V^−1^ s^−1^ for Bi_2_O_2_Se to ~15.44 *m*_0_^3/2^ cm^2^ V^−1^ s^−1^ for Bi_1.90_Ta_0.10_O_2_Se, revealing that Ta doping in Bi_2_O_2_Se can effectively optimize the electrical transport properties.

The *κ*_lat_ continuously decreases as the Ta content increases, reaching ~0.69 W m^−1^ K^−1^ for Bi_1.88_Ta_0.12_O_2_Se at 823 K (Figure 9d). The phonon mean-free-path (*l*_ph_) is calculated by the following Formula (6) [75,76] and plotted in Figure 9e as a function of Ta content.

(6) $$\kappa_{\mathrm{lat}}=\frac{1}{3}C_{v}v_{a}l_{\mathrm{ph}}$$

where, *C*_v_ and *v*_a_ represent the specific heat capacity per unit volume and average sound speed, respectively. A highly intense phonon scattering process and decrease of *κ*_lat_ in Ta-doped Bi_2_O_2_Se can be seen from monotonically reduced *l*_ph_ from ~11.9 Å for Bi_2_O_2_Se to ~9.9 Å for Bi_1.90_Ta_0.10_O_2_Se, which mainly results from that Ta substitution introduces multi-scale lattice defects, including the enormous defects, grain boundaries, and phase interfaces [68,77].

The carrier engineering and hierarchical microstructure by Ta doping remarkably enhance the *ZT* values in Bi_1.90_Ta_0.10_O_2_Se, reaching ~0.30 at 773 K, which is an increase of ~350% compared to pristine Bi_2_O_2_Se (Figure 9f).

## 4. Attempts to Realize a New Kind of *n*-Type Oxyselenide: Bi_6_Cu_2_Se_4_O_6_

**Figure 10:**
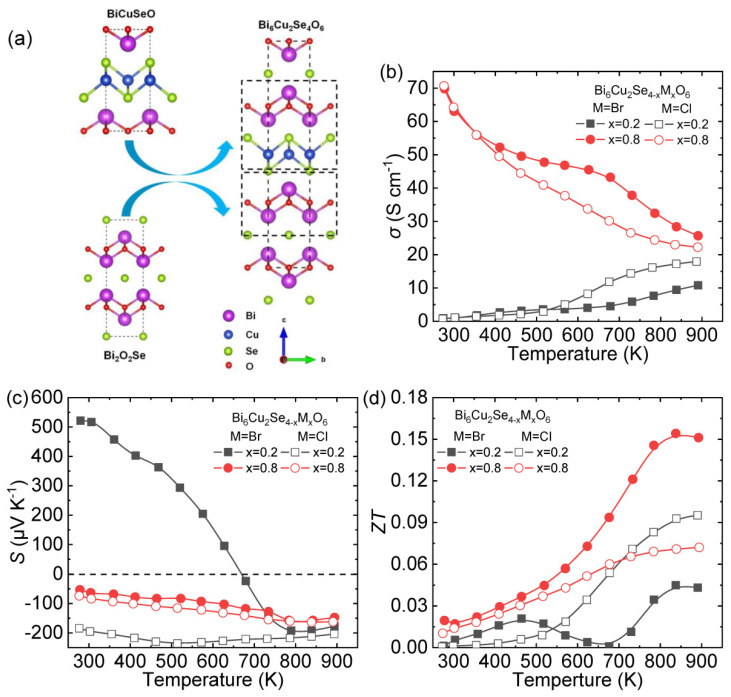
(**a**) The crystal structure of Bi_6_Cu_2_Se_4_O_6_. The temperature dependence of thermoelectric transport properties for Bi_6_Cu_2_Se_4−x_M_x_O_6_ (M = Cl/Br, *x* = 0.2/0.8): (**b**) electrical conductivity, (**c**) Seebeck coefficient, (**d**) *ZT*. Data shown in (**b**–**d**) were adopted from Reference [29]. Copyright 2019, WILEY-VCH Verlag GmbH & Co. KGaA, Weinheim.

Because of the strong phonon scattering caused by layered structure [9], the lone pair electrons of Bi^3+^ [78,79], and the local vibration of Cu^+^ [38], BiCuSeO exhibits inherent low thermal conductivity. Another well-known thermoelectric oxyselenide, Bi_2_O_2_Se reveals stable *n*-type transport properties due to Se vacancies [43,44]. To fully utilize the features of BiCuSeO and Bi_2_O_2_Se, a new type layered oxyselenide Bi_6_Cu_2_Se_4_O_6_ was synthesized through solid state reaction (SSR) with the 1:2 ratio of BiCuSeO and Bi_2_O_2_Se (Figure 10a) [28,29,30], and stable *n*-type conductive transports were observed in this system through halogen element doping [29].

### 4.1. Halogen Element Doping at Se Sites

The *σ* of Cl-doped Bi_6_Cu_2_Se_4_O_6_ is higher than Br-doped one at high temperature for the doping content *x* = 0.2. When the doping content *x* is increased to 0.8, the *σ* was significantly improved to ~70 S cm^−1^, and the Br-doped sample was better *σ* than the Cl-doped one at high temperature (Figure 10b). The Bi_6_Cu_2_Se_3.8_Br_0.2_O_6_ exhibits *p*-type semiconductor characteristics below 673 K and transfer to *n*-type with the temperature increasing; while Bi_6_Cu_2_Se_3.8_Cl_0.2_O_6_ has a negative *S* value within the entire temperature boundaries indicating that a small amount of Cl doping (*x* = 0.2) can achieve stable *n*-type semiconductor behavior (Figure 10c). When the doing content raises up to 0.8, the *S* of Cl/Br-doped samples has little difference, varying from ~−60 to −160 μV K^−1^. The maximum *ZT* value ~0.15 at 823 K is achieved in Bi_6_Cu_2_Se_3.2_Br_0.8_O_6_ (Figure 10c).

### 4.2. Transition Metal Element Doping at Bi Sites

**Figure 11:**
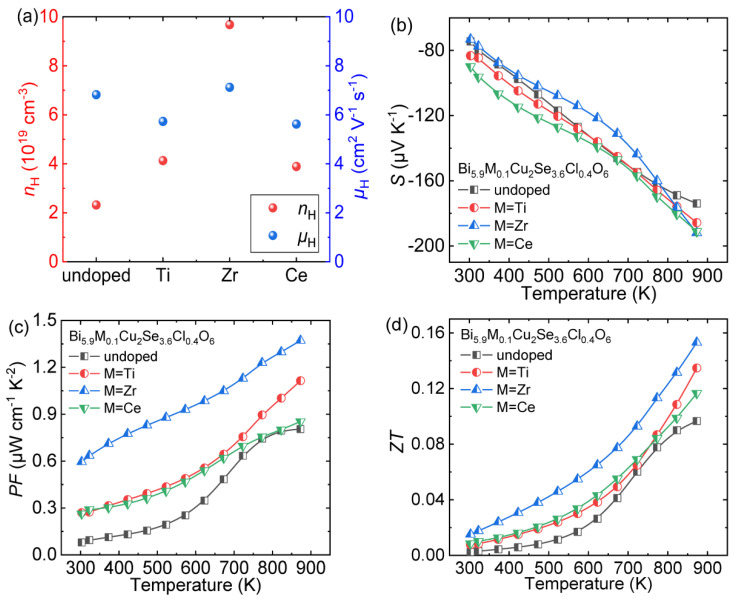
Thermoelectric transport properties of Bi_6_Cu_2_Se_3.6_Cl_0.4_O_6_ and Bi_5.9_M_0.1_Cu_2_Se_3.6_Cl_0.4_O_6_ (M = Ti, Zr, Ce): (**a**) carrier concentration (*n*_H_) and mobility (*μ*_H_) at room temperature, (**b**) Seebeck coefficient, (**c**) power factor and (**d**) *ZT*. Data shown in (**b**–**d**) were adopted from Reference [30]. Copyright 2021, Acta Materialia Inc.

Zheng et al. chose Bi_6_Cu_2_Se_3.6_Cl_0.4_O_6_ as the matrix and doped transition metal elements (Zr, Ti and Ce) at Bi sites to enhance its thermoelectric performance [30]. The introduction of transition metal elements can effectively increase the carrier concentration (*n*_H_) and maintain the carrier mobility (*μ*_H_; Figure 11a), thereby optimizing the electric conductivity (*σ*) of the matrix. The *S* of all doped samples remains negative throughout the entire temperature range, indicating the stable *n*-type semiconductor properties (Figure 11b). Thanks to the optimized *σ* and maintained *S*, the power factor (*PF*) is effectively enhanced (Figure 11c). Finally, due to the enhanced electrical transport properties and reduced thermal conductivity [30], the peak *ZT* value reached ~0.16 at 873 K in Bi_5.9_Zr_0.1_Cu_2_Se_3.6_Cl_0.4_O_6_ (Figure 11d), which is 60% higher than that in Bi_6_Cu_2_Se_3.6_Cl_0.4_O_6_ (~0.10 at 873 K).

As a new type of layered oxyselenide thermoelectric material, Bi_6_Cu_2_Se_4_O_6_ maintains the advantages of BiCuSeO and Bi_2_O_2_Se, such as lower-cost and nontoxic elements, better thermal and chemical stability. Moreover, Bi_6_Cu_2_Se_4_O_6_ can exhibit stable *n*-type semiconductor behavior by simple halogen doping and has intrinsic low thermal conductivity due to complex crystal structure. Thereby, Bi_6_Cu_2_Se_4_O_6_ is a new kind of *n*-type layered oxyselenide thermoelectric material with broad development prospects.

## 5. Summary and Perspective

In this short review, we introduced the latest accomplishments in *n*-type layered oxyselenide thermoelectric materials, including BiCuSeO, Bi_2_O_2_Se and Bi_6_Cu_2_Se_4_O_6_. For BiCuSeO, many strategies have been used to enhance the thermoelectric performance of *p*-type systems, but there are few studies on *n*-type BiCuSeO, and it is difficult to obtain stable *n*-type semiconductor behavior. For Bi_2_O_2_Se, carrier engineering, band engineering, microstructure design, etc., achieved performance enhancements of Bi_2_O_2_Se, but the *ZT* value is still limited to 0.4 [68]. Moreover, a new kind of promising *n*-type transport properties can be obtained in layered oxyselenide Bi_6_Cu_2_Se_4_O_6_ through halogen element doping. Apart from the advancements mentioned above, there is still room left for further research, such as make full utilization of the anisotropic transport properties of those compounds through texturing microstructure and crystals growth.

## Data Availability

The data of this study are available from the corresponding author upon reasonable request.
